# Panax notoginseng saponins alleviate damage to the intestinal barrier and regulate levels of intestinal microbes in a rat model of chronic kidney disease

**DOI:** 10.1080/0886022X.2022.2143378

**Published:** 2022-11-10

**Authors:** Jing Xie, Xin Ma, Yixuan Zheng, Nan Mao, Sichong Ren, Junming Fan

**Affiliations:** a Clinical Medical College of Chengdu University of Traditional Chinese Medicine, Chengdu, PR China; b Department of Nephrology No.1, the Affiliated Hospital of Chengdu University of Traditional Chinese Medicine, Chengdu, PR China; c Department of Nephrology, the First Affiliated Hospital of Chengdu Medical College, Chengdu, PR China; d Clinical Medical College of Chengdu Medical College, Chengdu, PR China

**Keywords:** Chronic kidney diseases, panax notoginseng saponins, intestinal flora, renal function, intestinal barrier

## Abstract

**Objectives:**

Chronic kidney disease (CKD) is a long-term condition characterized by poor prognosis and a high mortality rate. Panax notoginseng saponins (PNS) are the main active ingredient of the traditional Chinese herb *Panaxnotoginseng(Burk.)F.H.Chen*, which has been widely reported to have various pharmacological effects. Here, we examined the effect of PNS on renal function and the modulation of intestinal flora and intestinal barrier in a rat model of adenine-induced CKD.

**Methods:**

Adenine was used to establish a rat model of CKD, biochemical testing, histopathologic examination, ELISA, immunohistochemical assay, western blot assay, and fecal microbiota 16s rRNA analysis was used to test the effect of PNS on CKD rats.

**Results:**

Adenine induced a significant decrease in glomerular filtration rate, an increase in urinary protein excretion rate, and pathological damage to renal tissue in CKD rats. TNF-α, MCP-1, IL-1β, IL-18, TMAO, and endotoxin levels were increased in the blood of the model rats. Application of PNS countered the effects of adenine, restoring the above parameters to the level observed in healthy rats. In addition, activation of the inflammatory proteins NF-κB (p65) and NLRP3 and the fibrosis-associated proteins α-SMA and smad3 were inhibited in the kidneys of CKD rats. Furthermore, PNS promoted the expression of the tight junction proteins Occludin and ZO-1, increased SIgA levels, strengthened intestinal immunity, reduced mechanical damage to the intestine, was reduced levels of DAO and D-LA. Our data suggest PNS may delay CKD by restoring gut microbiota, and through the subsequent generation of a microbial barrier and modulation of microbiota metabolites.

**Conclusions:**

In conclusion, PNS may inhibit the development of inflammation and fibrosis in the kidney tissue through regulation of intestinal microorganisms and inhibition of the activation of pro-inflammatory and pro-fibrotic proteins in the kidney.

## Introduction

Chronic kidney disease (CKD) is a long-term condition characterized by a slow and gradual decline in kidney function, and patients suffer irreversible damage to the kidney and multi-system involvement, posing a serious threat to their life [1,2]. Although the development of renal replacement therapies such as hemodialysis, peritoneal dialysis, and kidney transplantation has led to better treatments for patients with end-stage CKD [3,4]. However, alternative therapies remain costly and are not suitable for patients with early or mid-stage CKD and mild to moderate CKD [5,6]. Thus, seeking and developing high-effect, low-poison and cheap natural medicine for the treatment of CKD has become an international research hotspot.

In recent years, there have been increasing reports on the role of gut flora in the development and progression of CKD, and several studies have confirmed that dysbiosis of the gut microbiota may be a key factor in the pathogenesis of kidney disease [7,8]. Dysbiosis of the intestinal flora may lead to the passage of toxins and pathogens through the intestinal barrier [9]. The original structure of the intestinal flora is broken, harmful microorganisms proliferate while beneficial flora is suppressed, and toxins of all kinds are produced in large quantities, even entering the bloodstream through the intestinal barrier and flowing to all systems of the body [10]. Furthermore, exposure of the body to endotoxins causes a systemic inflammatory response and oxidative stress and further induces and accelerates kidney damage [11]. In addition, except for intestinal flora, intestinal flora metabolites are also considered to be important in regulating the vital activities and metabolism of the body and are involved in the development of many diseases, including diabetes or CKD [12,13]. Panax notoginseng saponins (PNS) is the main active ingredient of the traditional Chinese herb *Panaxnotoginseng(Burk.)F.H.Chen*, which has been widely reported to have pharmacological effects such as anti-myocardial ischemia, anti-myocardial infarction, anticoagulation, lipid regulation, hypotension, anti-inflammation, and antioxidant [14,15]. Previous studies have reported that PNS protects the kidney from diabetes by up-regulating silent information regulator 1 and activating antioxidant proteins in rats [16]. More importantly, PNS can modulate the gut microbiota to promote thermogenesis and beige adipocyte reconstitution through leptin-mediated AMPKα/STAT3 signaling to cure diet-induced obesity [17]. And PNS also has a preventive effect on colitis-associated colorectal cancer development [18]. More importantly, previous studies have shown that PNS attenuates cisplatin-induced nephrotoxicity *via* the HIF-1α and mitochondrial apoptosis [19,20]. Notoginsenoside r1 attenuates renal ischemia-reperfusion injury in rats [21]. A rat model of CKD induced by adenine is widely used in basic research, and the model can replicate some of the clinical signs of CKD [22]. We hypothesized that this animal model could be well suited for analyzing pathophysiological mechanisms in CKD. This study aimed to determine the effect of PNS on renal function in CKD rats and to examine the regulation of intestinal flora and changes in proteins associated with the development of renal inflammation.

## Materials and methods

### Animals and grouping

Forty-two male specific pathogen free (SPF SD) rats were purchased from the Animal Center of DASHUO [SCXY (Chuan) 2020-034]. Four-week-old rats weighing 150 ± 20 g, acclimatized to the environment for one week. The rats were randomly divided into blank group, model group, positive drug (valsartan, Novartis China) treatment group, and PNS low-, medium-, and high-dose groups. Except for the blank group, the other groups were given adenine (200 mg/kg daily) by gavage for 28 consecutive days to establish a CKD rat model. After finishing the CKD rat model, PNS was administered at 40 mg/kg, 80 mg/kg, and 160 mg/kg, and the positive drug at 13 mg/kg once daily by gavage. On day 29, the blank and model groups were given an equal volume of saline during 28 days of continuous administration of PNS (Chengdu Pufei De, China) and positive drugs. After finishing the experiment, the animals were intraperitoneally injected with 1% sodium pentobarbital (50 mg/kg) and sacrificed to collect serum, colon, ileum, and kidney for the next experiment. Before sacrificed, rats were housed in individual metabolic cages (Laboratory Products, Seaford, DE) to collect urine for 24 h. Tissues, urine and blood were collected after execution of the rats for the next study. All procedures in this study were under the requirements of the Animal Experimental Committee and the Ethics Committee of the Chengdu Medical College (Ethical number: 20211553 A). The work in this study have been carried out in accordance with the ARRIVE Guidelines for reporting *in vivo* animal experiments (http://www.nc3rs.org.uk/page.asp?id=1357).

### Biochemical testing

Urine was collected for 24 h using metabolic cages (VWR, NALGENE Single-Mouse Metabolic Cage). Blood was collected from the abdominal aorta of rats and the supernatant was separated by centrifugation at 4 °C for 10 min for biochemical testing. The urine of rats was collected for 24 h for biochemical assay. The creatinine and urea (UREA) nitrogen levels were measured using a fully automatic biochemical analyzer (Beckman, German) and matching kits. Creatinine (UCr and SCr) and urine albumin related to kidney function, are measured by ELISA kits (C035-2-1, Nanjing Jiancheng Bioengineering Research, China). Calculation of glomerular filtration rate (GFR) and urine albumin to creatinine ratio (ACR) from measured values. The equations used were as follows: GFR = (Ucr × 24 h urine volume)/(Scr × 24 h), ACR (an indicator of urine protein levels) = urine albumin/Ucr.

### Histopathologic examination

The kidney, ileum, and colon tissue of rats were fixed with paraformaldehyde for 24 h, routinely paraffin-embedded sections were stained with hematoxylin & eosin (Servicebio, Wuhan, China), and the histological differences were observed under the microscope. Five fields of view were randomly selected from the transverse section of the tissue under a 100× and 40× microscope.

### Enzyme-linked immunosorbent assay (ELISA)

Blood was collected from rats in each group and centrifuged at 1500 rpm for 10 min to obtain the serum. In the first part of the experiments, the levels of pro-inflammatory cytokines including LPS (Cusabio, Wuhan, China), monocyte chemoattractant protein-1 (MCP-1; Abcam, UK), TNF-α (Beyotime, China), and IL-1β (Abcam, UK) were determined by ELISA according to the manufacturer’s instructions. In the second part of the experiments, the levels of trimethylamine oxide (TMAO, Zci Bio, Shanghai, China), secretory IgA (SIgA, Zci Bio, Shanghai, China), diamine oxidase (DAO, Zci Bio, Shanghai, China), D-LA (D-lactic acid, Zci Bio, Shanghai, China), Occludin (Zci Bio, Shanghai, China), and tight junction protein 1 (ZO-1, Zci Bio, Shanghai, China) were measured by ELISA according to the manufacturer’s instructions. The OD value of each well was immediately read at 450 nm.

### Immunohistochemical assay

Four-micrometer paraffin sections of kidney were prepared, and then incubated with primary antibody, including TNF-α (Abcam, UK), IL-1β (Abcam, UK), IL-18 (Abcam, UK), and α-SMA (Abcam, UK), overnight at 4 °C, and then incubated with the appropriate amount of biotinylated goat anti-rabbit IgG secondary antibody (s0002, Affinity) for 30 min at 37 °C. Finally, the sections were stained with DAB (AR1025, BOSTER, China) and re-stained with hematoxylin. The positive expression of TNF-α, IL-1β, and α-SMA protein was observed under an optical microscope, and the positive cells were stained yellow or brown. Image-Pro Plus6.0 image analysis software was used to count positive cells.

### Western blot assay

Kidney tissues were lysed with RIPA buffer to obtain total protein. After the protein concentration was determined by the BCA protein detection kit (Abcam, UK), the total protein was performed with sodium dodecyl sulfate (SDS) polyacrylamide gel electrophoresis and electrotransfer. Then, the membrane is sealed with 5% completely skimmed milk powder, add the diluted primary antibody solution (NF-κB p65, 6535-1-Ig, Proteintech), (NLRP3, DF7438, Affinity), (caspase-1, AF5418, Affinity), (α-SMA, AF1032, Affinity), (smad3, AF6362, Affinity), (p-smad3, AP0727, Abclonal) and incubate the membrane at 4 °C for 12 h. Next, the membrane was washed with PBST (every 15 min) four times, then incubated with the diluted secondary antibody at room temperature for 2 h to enhance the chemiluminescence detection system immune signal. β-actin as a reference and Image J software was used for optical density analysis.

### Real-time quantitative reverse transcription PCR

TRIzol kit (TAKARA, Jap) was used to extract total RNA from the kidney, and the concentration and purity of RNA were determined by UV spectrophotometry. When the ratio of A260/A280 is between 1.8 and 2.0, the sample is considered qualified. After reverse transcription of total RNA into cDNA, PCR amplification was performed by RT-qPCR kit steps (TAKARA, Jap). Genes Forward primer Reverse primer NLRP3: 5′-AGACAGCCTTGAAGAGGAGTGGATAG-3′, 5′-AACCTGCTTCTCACATGCCTTCTG’. Smad3: 5′-AGTGGTGCGAGAAGGCGGTCAAGA-3′, 5′-CGTAACTCATGGTGGCTGTGCAGGTC-3′. Caspase-1: 5′-CCTGGACCGAGTGGTTCCCTCAAGT-3′, 5′-GGCAAGACGTGTACGAGTGGGTGTTT-3′. α-SMA: 5′-TCCTGACCCTGAAGTATCCGATAGAAC-3′, 5′-CGTTATAGAAGGAGTGGTGCCAGATC-3′. NF-κB: 5′-GTCACCACTGTTGCGACCCTTG-3′, 5′-GCTTCAGCTTGGAAAAGGCATCTTC-3′. β-actin: 5′-GGGAAATCGTGCGTGACATT-3′, 5′-GCGGCAGTGGCCATCTC-3′. With β-actin as a reference, the 2^−ΔΔCt^ method was used to calculate NF-κB (p65), NLRP3, caspase-1, α-SMA, and smad3 mRNA expression.

### Fecal microbiota 16S rRNA analysis

DNA was extracted from approximately 0.25 g of fecal samples using the QIAamp Fast DNA Stool Mini Kit (Qiagen, CA, USA) according to the manufacturer’s instructions. Then PCR was performed on the 16SV3-V4 area of DNA Amplification, primer sequence: forward primer is 5′- ACTCCTACGG GAGGCAGC AG-3′, reverse primer is 3′-GGACTACHVGGGTWTCTAAT-5′. Take the diluted gene group DNA as a template, use KAPA HiFiHotstar ReadyMix PCR. The kit high-fidelity enzyme performs PCR to ensure the accuracy and high Effectiveness. The sequencing library was prepared using TruSeq Nano DNA LT Library Prep Kit from Illumina.

### Statistical analysis

GraphPad Prism 8.0 software was used for performing the statistical analysis. Data were expressed as standard error of the mean. One-way analysis of variance (one-way ANOVA) was used for comparison of more than two groups, and a T-test was used for expression differences between the two groups. *p* < .05 was considered statistically significant.

## Results

### PNS improves renal function in adenine-induced CKD rats

**Figure 1.**
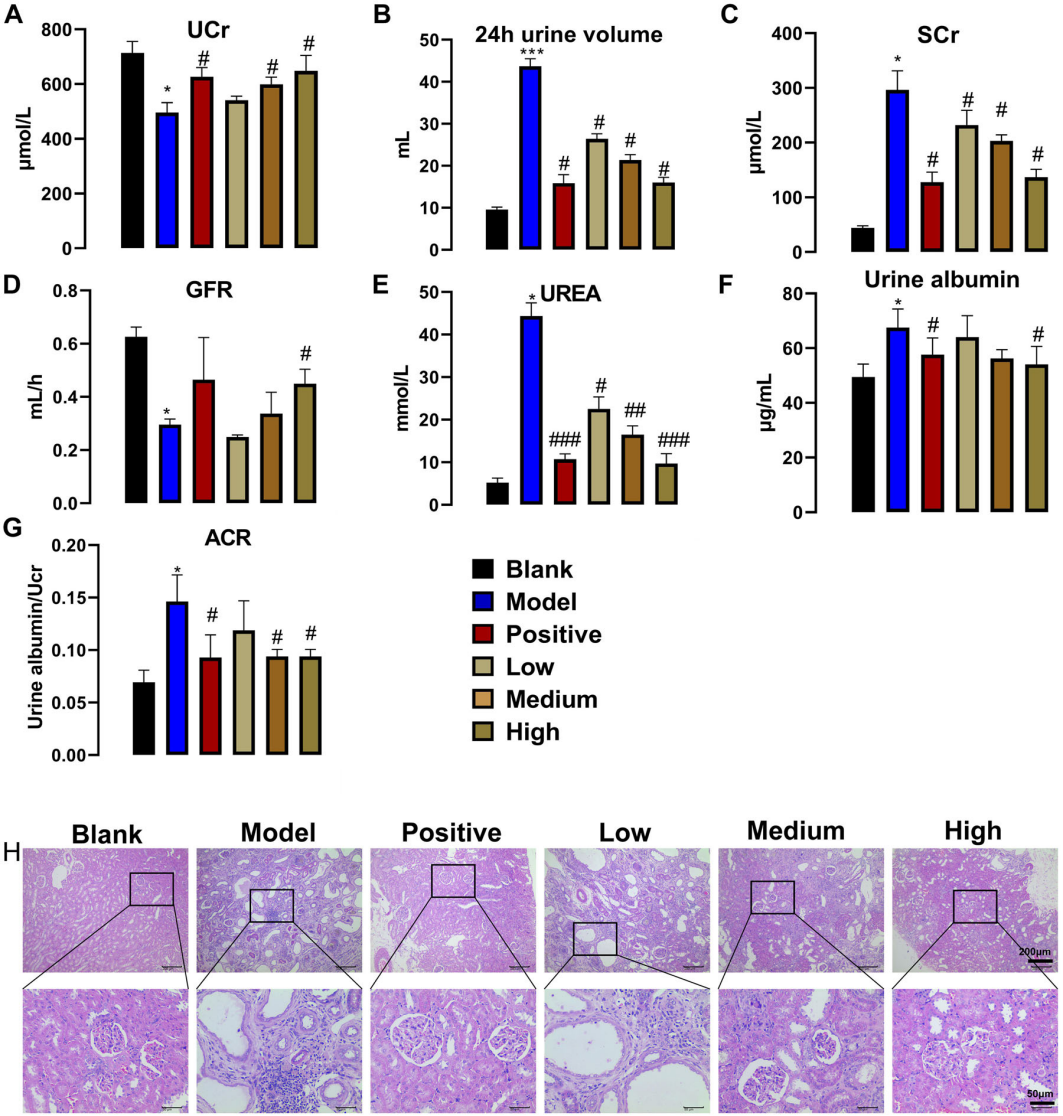
PNS concentration-dependently improved renal function and kidney tissue pathology in rats (*n* = 7). (A) Ucr. (B) 24 h urine volume. (C) SCr. (D) GFR. (E) UREA. (F) Urine albumin. (G) excretion protein. (H) H&E-stained kidney tissue after administration of PNS were observed at 100× and 400×. Ucr, SCr, UREA, and urine albumin were detected by biochemical testing, and 24 h urine volume was measured, and the above data were used to calculate GFR and excretion protein according to the formula. Data are mean ± SEM, *compared with blank group, *p < .05 and ***p < .001, ^#^compared with model group, ^#^*p* < .05, ^##^*p* < .01, ^###^*p* < .001.

Male adenine-induced CKD SD rats were used to examine the effects of PNS on renal function and renal histopathology. As shown in Figure 1(A–D), compared with the blank group, UCr was significantly reduced by the adenine-induction effect, while 24 h urine volume and SCr were increased. Compared with the model group, treatment with the positive drug valsartan resulted in increased levels of UCr and a significant decrease in 24 h urine volume and SCr. In addition, PNS has a dose-dependent effect on the regulation of UCr, 24 h urine volume, and SCr. As for GFR, the model group showed a significant reduction in GFR compared to the blank group, while the positive drug had some restorative effect and PNS restored GFR in a dose-dependent manner, with significant effects at higher doses. As shown in Figure 1(E), adenine induced significant increases in UREA in CKD rats, while treatment with the positive drug significantly suppressed UREA. Similarly, PNS treatment concentration-dependently ameliorated these differences. As shown in Figure 1(F,G), compared with the blank group, the urine albumin content in the model group increased, while the positive drug and high-dose PNS could reverse the results caused by adenine. Meanwhile, both the positive drug and PNS can regulate the urine protein exclusion rate of CKD rats induced by adenine. As shown in Figure 1(H), adenine-induced CKD rats showed marked tubular dilatation, fibrous tissue hyperplasia, and inflammatory cell infiltration. In contrast, the kidney tissues of the rats treated with positive drugs had intact peritoneal membranes, normal tubular epithelial cell morphology, no obvious fibrous tissue hyperplasia, and inflammatory cell infiltration in the renal interstitium. In addition, PNS has a dose-dependent resistance to this adverse reaction caused by adenine. Taken together, these results indicated that administration of PNS improved renal function and kidney tissue pathology in CKD rats.

### PNS regulates cellular factors related to CKD in serum

**Figure 2.**
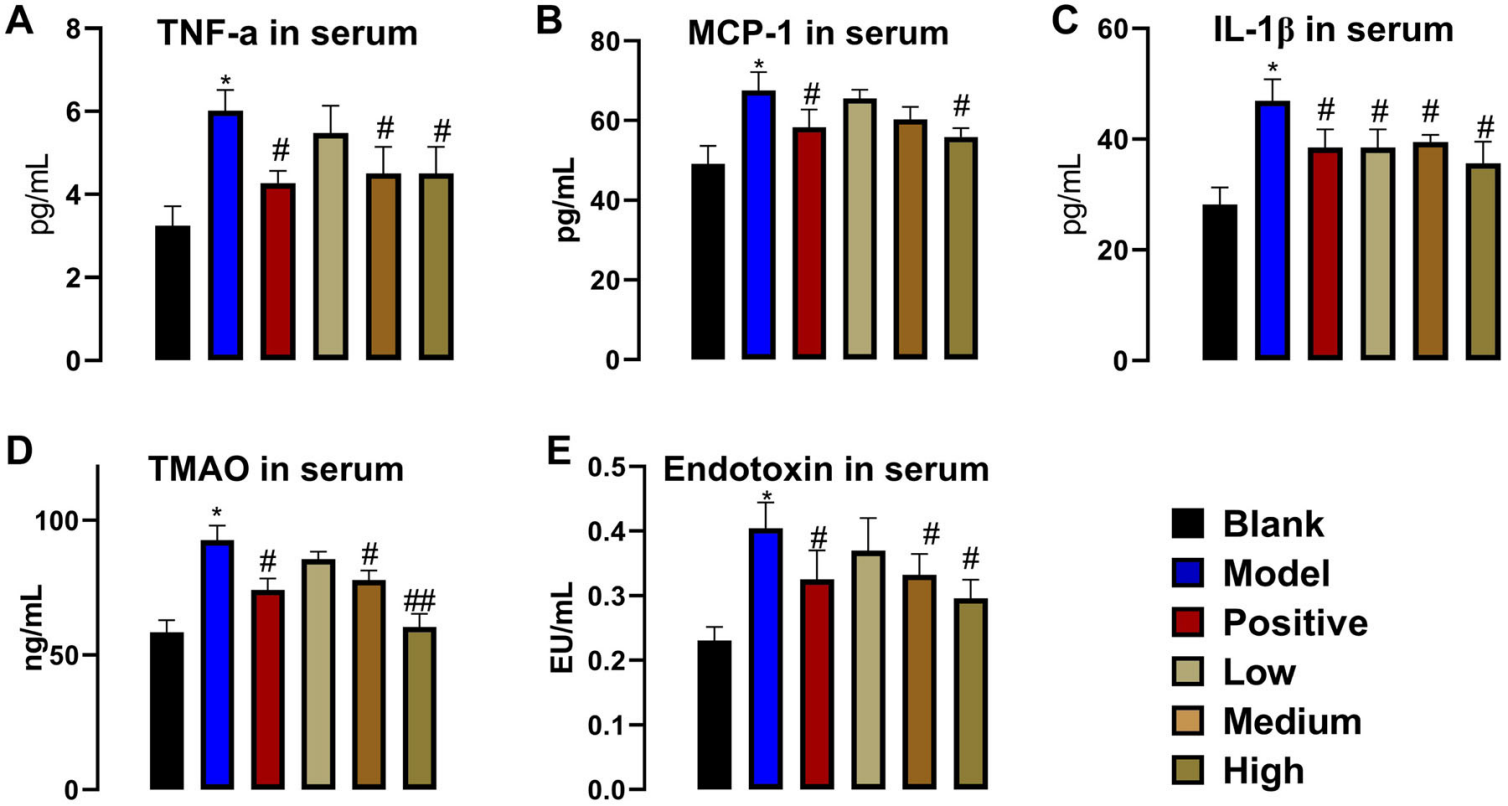
PNS concentration dependently affects cytokines in the serum of CKD rats and intestinal flora metabolism-related product TMAO (*n* = 7). (A) TNF-α. (B) MCP-1. (C) IL-1β. (D) TMAO. (E) endotoxin (LPS). ELISA assay for the determination of serum TNF-α, MCP-1, IL-1β, TMAO, and LPS. Data are mean ± SEM, * compared with blank group, *p < .05; ^#^ compared with model group, ^#^*p* < .05.

Serum cytokines TNF-α, MCP-1, IL-1β, endotoxin are closely associated with the development of CKD, and up-regulation of these cytokines is a marker of progression in CKD. TMAO (a metabolism-related product of intestinal flora) has been shown to impair kidney function and worsen the course of CKD. As shown in Figure 2(A–D), compared with the blank group, the serum levels of TNF-α, MCP-1, IL-1β, and endotoxin in the model group increased significantly, while the increased cytokines decreased under the action of positive drugs. In addition, compared with the model group, these cytokines were reduced under the action of PNS with a dose-dependent relationship. As shown in Figure 2(E), TMAO increased significantly in the model group, the serum TMAO content decreased under the action of positive drugs, and these cytokines showed a dose-dependent decrease under the action of PNS. These results indicate that the decreased content of pro-inflammatory cytokines in peripheral blood of CKD rats under the action of PNS may reduct renal tissue lesions. And, TMAO has also been significantly down-regulated, suggesting that PNS may affect the metabolism of intestinal flora.

### PNS regulates proteins related to CKD rats in kidney

**Figure 3.**
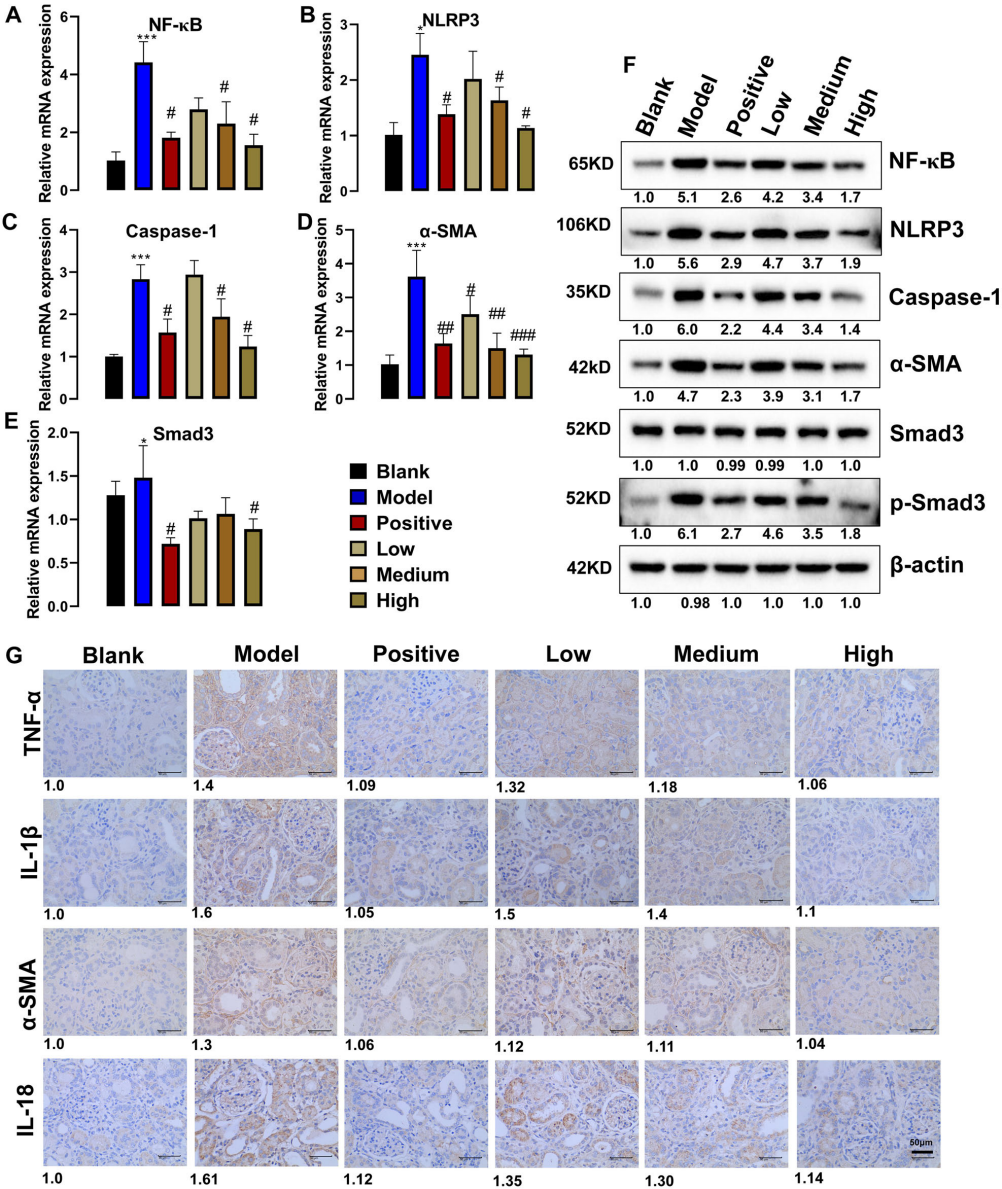
PNS inhibits the activation of proteins associated with inflammation regulation (*n* = 7). (A–E) The mRNA level of NF-κB (p65), NLRP3, caspase-1, α-SMA, and smad3 were detected by RT-qPCR. (F) The proteins expression of NF-κB (p65), NLRP3, caspase-1, α-SMA, smad3, and p-smad3 were detected by western blot. (G) Immunohistochemistry for TNF-α, IL-1β, IL-18, and α-SMA. Data are mean ± SEM, * compared with blank group, *p < .05 and ***p < .001; ^#^ compared with model group, ^#^*p* < .05, ^##^*p* < .01, ^###^*p* < .001.

To further explore the therapeutic mechanisms of PNS against CKD, NF-κB (p65), NLRP3, caspase-1, α-SMA, and smad3, which are associated with inflammation and proliferation, were examined in renal tissue. As shown in Figure 3(A–E), compared with the blank group, mRNA levels of NF-κB (p65), NLRP3, caspase-1, α-SMA, and smad3 were significantly increased under adenine induction, while these increased mRNAs tended to decrease after positive drug treatment. Moreover, PNS had a similar effect to the positive drug and could inhibit these up-regulated mRNAs. As shown in Figure 3(F), the expression of these proteins was consistent with the results of the mRNAs, with NF-κB (p65), NLRP3, caspase-1, α-SMA, and p-smad3 all appearing up-regulated in the model group, while the positive drugs and PNS were able to down-regulate these activated proteins. As shown in Figure 3(G), compared with the blank group, TNF-α, IL-1β, IL-18, and α-SMA increased significantly in the adenine-induced CKD model, while the positive drugs could inhibit TNF-α, IL-1β, IL-18, and α-SMA. Furthermore, TNF-α, IL-1β, IL-18, and α-SMA showed a dose-dependent decrease in response to PNS treatment. These results suggested that PNS inhibits the activation of pro-inflammatory and fibrotic proteins and thus inhibits inflammatory factors and renal fibrous tissue proliferation.

### PNS regulates the intestinal barrier

**Figure 4.**
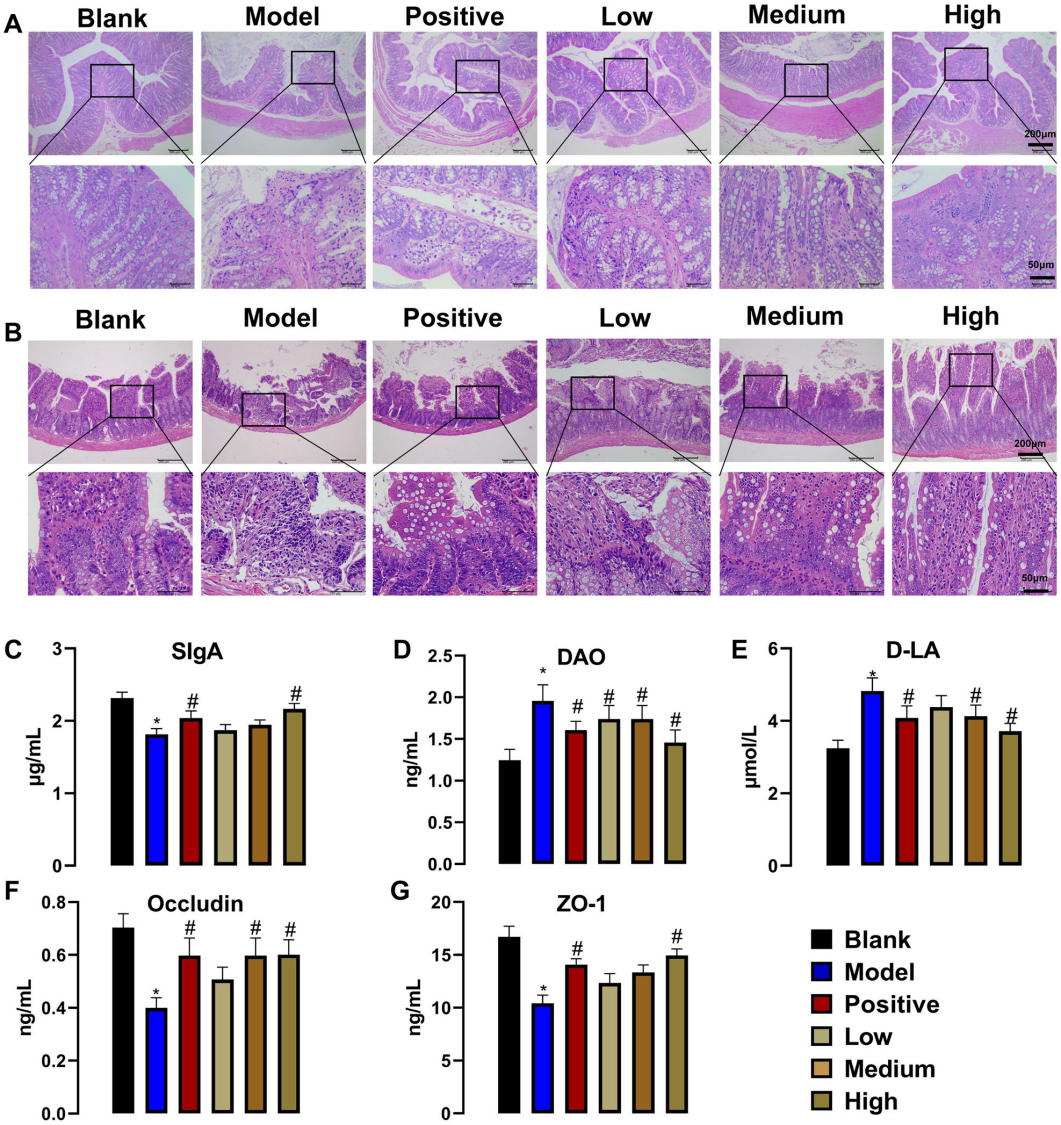
PNS inhibits the activation of proteins associated with inflammation regulation (*n* = 7). (A) H&E-stained colonic tissue after administration of PNS at 100× and 400×. (B) H&E-stained ileum tissue after administration of PNS at 100× and 400×. SIgA. (D) DAO. (E) D-LA. (F) Occludin. (G) ZO-1. ELISA assay for the determination of SIgA, DAO, D-LA, Occludin, and ZO-1. Data are mean ± SEM, * compared with blank group, *p < .05; ^#^ compared with model group, ^#^*p* < .05.

The decrease of intestinal barrier function and the increase of intestinal permeability in CKD may be important reasons for the increase of serum endotoxemia and TMAO. As shown in Figure 4(A), compared with the blank group, the colon tissue of the model group was locally damaged, the mucosal epithelium was missing after necrosis and exfoliation, the intestinal glands within the lamina propria degenerated and necrotic, the morphological structure of the cup-shaped cells was blurred, cellular debris and lymphocyte aggregates were visible in the necrotic area. Compared with the model group, the colon tissue of the positive drug group was slightly damaged, and the mucosal epithelium was arranged more neatly. Meanwhile, PNS can relieve colonic mucosal damage and has a dose-effect. In addition, the model group showed some damage to the ileal tissue, and these lesions were reduced to some extent by the PNS and positive drugs (Figure 4(B)). As for intestinal barrier-related factors SIgA, DAO, D-LA, Occludin, and ZO-1, compared with the blank group, DAO and D-LA increased significantly in the model group but decreased under the action of positive drugs and PNS. In addition, SIgA, Occludin, and ZO-1 were significantly reduced in the model group, with some recovery in the presence of positive drugs. Similarly, PNS promoted levels of SIgA, Occludin, and ZO-1, with better effects at higher doses (Figure 4(C–G)). In general, PNS may affect the occurrence and development of CKD by regulating the intestinal barrier.

### Analysis of intestinal flora in CKD rats regulatde by PNS

The disturbance of the intestinal barrier function in patients with CKD is often accompanied by disturbances in the intestinal flora, and disturbances in the intestinal flora are increasingly recognized as part of the pathogenesis of CKD.

**Figure 5.**
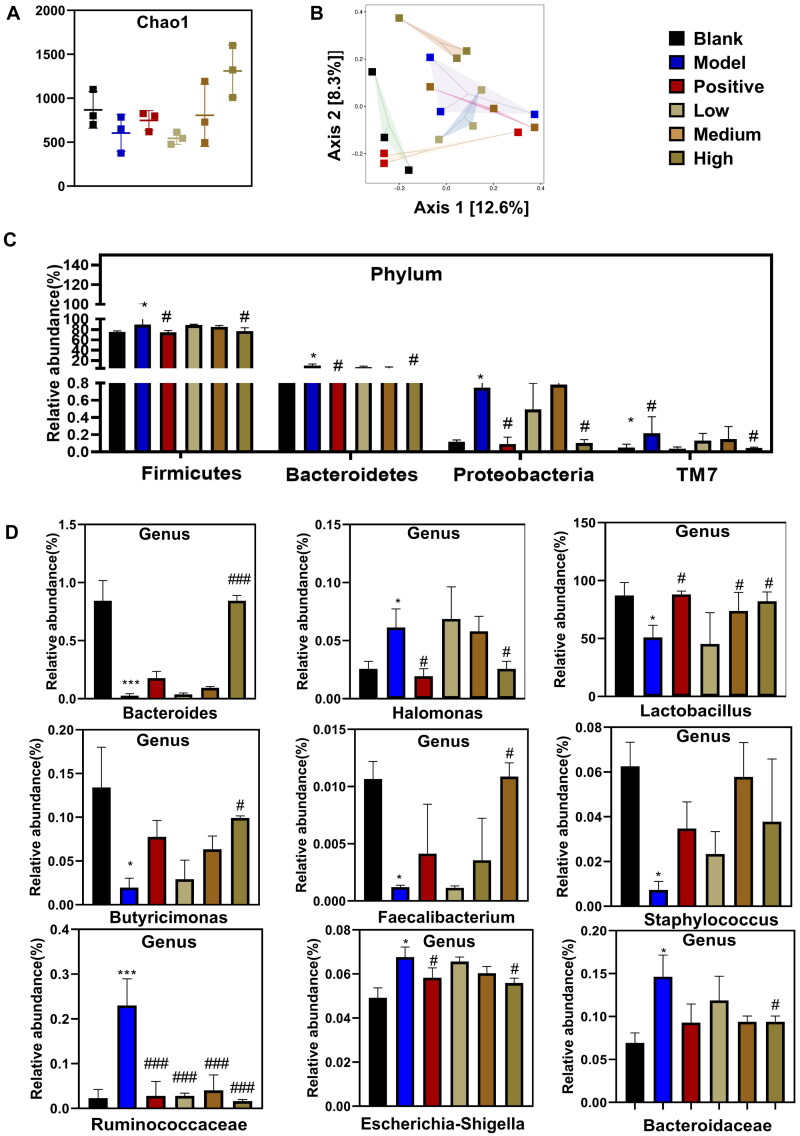
Effect of PNS on the intestinal microflora (*n* = 7). (A) The abundance of intestinal flora at the phylum level. (B) The abundance of intestinal flora at the genus level. (C) Grouped box plot of alpha diversity index. (D) Beta diversity analysis sample ranking chart for PCoA analysis. Evaluation of the effect of PNS on intestinal flora at the phylum and genus levels and changes in intestinal flora diversity by 16S rRNA sequencing. Data are mean ± SEM, *compared with blank group, *p < .05 and ***p < .01; ^#^ compared with model group, ^#^*p* < .05 and ^###^*p* < .001.

As shown in Figure 5(A), Chao1 was considered to fully assess the alpha diversity of the microbial community, the alpha diversity of the model group decreased, and the positive drugs had little effect on alpha diversity, while the high-dose PNS could enrich the microbial composition. As shown in Figure 5(B), the distribution of PCoA between the model and high dose of PNS groups indicated significant discrete distribution, while the spatial distance difference between the blank and positive group is closer. In addition, the proximity of the model and high-dose PNS groups within the group suggests that the species composition of the groups is structurally similar. As shown in Figure 5(C), at the gate level, adenine promoted the growth of *Firmicutes*, *Bacteroidetes*, *Proteobacteria*, and *TM7*, while positive drugs and high doses of PNS had some inhibitory effect on these microorganisms. As shown in Figure 5(D), Some bacteria in the fecal samples, such as *Bacteroides*, *Halomonas*, *Lactobacillus*, *Butyricimonas*, *Faecalibacterium*, *Staphylococcus*, *Ruminococcaceae*, *Escherichia-Shigella*, and *Bacteroidaceae*, showed dramatical changes at the genus classification level after the induction of adenine. And there were significant differences between the adenine-induced model CKD rats and the high dose of the PNS group. The results showed that PNS affect the progression of CKD by affecting the intestinal flora.

## Discussion

CKD is caused by the end-stage development of many kidney diseases when the kidneys lose their basic functions, manifested by systemic metabolic dysfunction, abnormal urine and blood indicators, and a poor prognosis [1,23]. Therefore, replication of stable animal models of CKD and selection of positive drugs were key aspects of the search for drugs to combat CKD. Adenine-induced nephropathy is an established model of CKD in rodents. Upon Adenine ingestion, pathological changes are observed in kidney tissues, and renal function is reduced, as indicated by UREA and CREA. These changes are close to the clinical signs of patients with CKD [22,24]. Valsartan was the most commonly applied drug in the treatment of CKD with reduction of urinary protein [25]. In this study, we successfully modeled CKD rats, as evidenced by an increase in 24 h urine volume, a decrease in GFR, and an increase in urinary protein elimination rate. The adenine-induced CKD rat model has been extensively studied in rat models, and the metabolic abnormalities caused by long-term administration of adenine are similar to the clinical and pathological changes in human chronic renal failure [26,27]. We observed that PNS improved renal function and renal histopathological damage in CKD rats in a dose-dependent manner, and at high doses had similar effects to the positive drug valsartan.

Previous studies have reported alterations in the gut microbiota in animal models of adenine-induced CKD compared with normal mice [28]. Under normal conditions, there is a fairly stable proportional relationship between the intestinal flora, which bind to the intestinal mucosa and form a regular membrane flora in an adhesive or chimeric manner, forming the microbial barrier of the intestine [29,30]. In addition, intestinal epithelial tight junctions play an important role in maintaining the permeability and integrity of the intestinal mucosa and are an essential component of the mechanical barrier of the intestine [31]. Moreover, elevated DAO and D-LA also imply damage to the intestine [32]. In the nephrotic model, there is a significant decrease in tight junction proteins (Occludin and ZO-1) and an increase in the permeability of the intestinal wall, leading to an easier passage of pathogens, endotoxins, or other harmful substances from the intestinal tract into the circulation. Moreover, SIgA is the main immunoglobulin of the intestinal mucosal immune barrier with the biological function of the immune barrier, preventing the invasion of intestinal pathogens, and reducing the inflammatory response of the intestine [33,34]. Previous studies have shown that intestinal barrier dysfunction increases intestinal permeability, and then harmful substances can pass through the intestinal mucosa, and small intestinal permeability has been used to quantify damage to the intestinal mucosal barrier [35,36]. This study showed that PNS promoted the repair of intestinal tissue damage and up-regulated intestinal tight junction proteins in CKD rats. The return of intestinal SIgA, DAO, and D-LA to blank group levels under PNS means that the intestinal barrier is restored, and it is more difficult for endotoxins or other harmful metabolites of the intestinal flora to cross the intestinal wall and enter the circulation.

Gut microbiota is the normal microbial community in the human intestinal tract, which plays a variety of physiological roles such as mechanical barrier, biological barrier, and immune barrier to the host [37]. Kikuchi et al. [38] found in a 5/6 nephrectomy model that serum urotoxins correlated with the abundance of *Clostridium* and *Bacteroides* in the intestinal flora. Yoshifuji et al. [39] conducted an in-depth analysis of the species composition of enteric bacteria in nephropathy and found that the most significant reduction in the model group was in the *Lactobacillidae family*. Currently, various data are available to support the imbalance in the number and species of intestinal flora in CKD [40,41]. For example, at the species and genus levels, an increase in the abundance of *Allobaculum*, *Escherichia_Shigella*, *Clostridium_sensu_stricto*, *Bacte-roides*, *Parasutterella*, *Ruminococcus*, *Blauti*a, and *Enter-orhabdus* and the decrease of *Lactobacillus* and *Bifido-bacterium* [40,41]. *Bacteroides* are significantly reduced in ESRD patients. Intestinal bacteria in patients with end-stage renal disease (ESRD) is significantly reduced, with enrichment of *Bacteroides* in the genus and a reduction in the abundance of some butyrate-producing bacteria including *Roseburia*, *Faecalibacterium*, *Clostridium*, *Coprococcus*, and *Prevotella* [42]. In the present study, PNS modulated intestinal flora diversity in CKD rats, restoring differentially present flora at the phylum and genus levels. The restored intestinal flora may delay CKD by establishing a microbial barrier and regulating the metabolites of the flora.

Changes in the intestinal flora cause changes in the metabolites produced by the intestinal bacteria and also in the bloodstream from intestinal sources [43]. Lipopolysaccharide (LPS) is a component of the outer membrane of *Gram-negative bacteria* and a major component of endotoxin, which is present in high plasma levels in patients with CKD and contributes to the inflammatory response of the host [44,45]. In addition, TMAO is considered to be the most important nephrotoxic metabolite of intestinal microorganisms [46]. Previous studies have shown that TMAO can induce interstitial nephritis [47], and TMAO plays a critical role in protecting renal cells from hyperosmotic stress [48,49]. The dysbiosis of the intestinal flora and the increased permeability of the intestinal barrier cause endotoxins and bacterial metabolic nephrotoxins to enter the circulation and the kidneys, leading to systemic and local inflammation of the kidneys and aggravating kidney disease [50]. In this study, the levels of TNF-α, MCP-1, IL-1β, TMAO, and endotoxin in the blood of CKD rats were all increased to varying degrees. These pro-inflammatory factors cause activation of the renal tissue inflammation-related proteins NF-κB (p65) and NLRP3 *via* the circulation, further affecting the development of renal tissue inflammation. Activation of the fibrosis-associated proteins α-SMA and smad3 leads to interstitial fibrosis, which affects the function of the renal tissue. Previous studies have shown that NF-κB (p65) can enter the nucleus to initiate inflammatory factor transcription and NLRP3 activation stimulates caspase-1 to produce precursors of IL-1β and IL-18 [51,52]. Macrophage and monocyte chemokine MCP-1 can activate and chemotactic monocytes to sites of inflammation [53]. In addition, TMAO induces α-SMA activation to induce smad3 phosphorylation to promote the development of renal fibrosis [54]. Taken together, PNS modulates metabolites of the intestinal flora, reduces circulation in the blood, inhibits activation of inflammatory-related proteins in renal tissue, and reduces inflammatory damage and interstitial fibrosis in renal tissue.

## Conclusions

**Figure 6.**
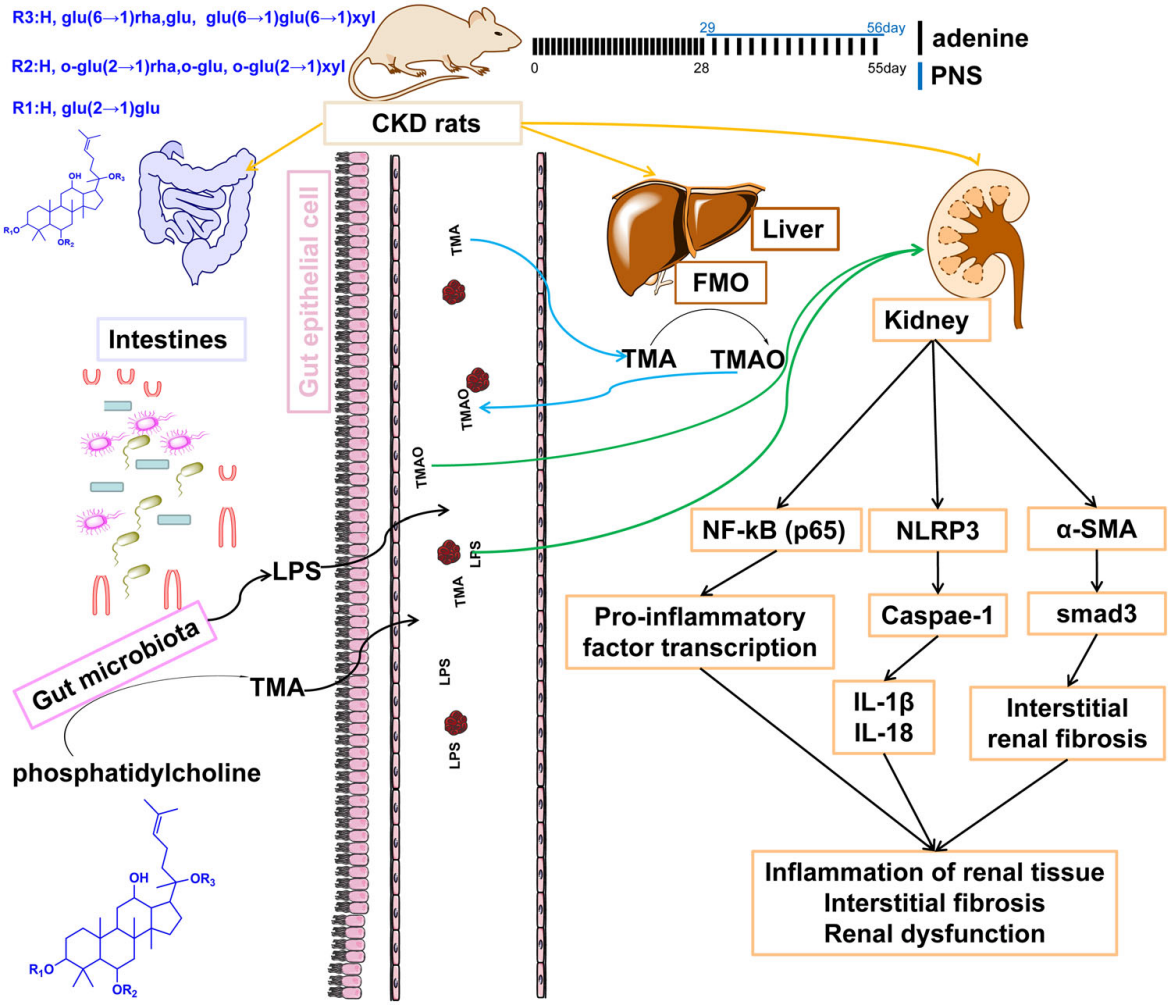
Schematic diagram of the protective mechanism of PNS against CKD. PNS protects the kidneys of CKD rats by modulating the intestinal barrier and metabolites of the intestinal microbiota. Phosphatidylcholine is fermented by the intestinal flora to TMA via the portal vein to the liver where it is oxidized by flavin-containing monooxygenase to TMAO. TMAO and LPS stimulate the development of inflammation and induce interstitial fibrosis in the kidney via circulation.

The results of this study found that PNS significantly improved renal function in adenine-induced CKD rats and down-regulated the pro-inflammatory factors TNF-α, IL-18, and IL-1β in renal tissues. The mechanism may modulate the composition of the intestinal microflora and thus repair the intestinal barrier to reduce the production of TMAO, a metabolism-related product of the intestinal flora. Meanwhile, PNS may inhibit NF-κB and NLRP3 activation to suppress the production of pro-inflammatory factors in renal tissue as well as inhibit the expression of the renal fibrosis proteins α-SMA and smad3 (Figure 6). Our findings provide a theoretical basis for pre-clinical studies of PNS as a potential treatment for CKD. One concern about the findings was that PNS is a mixture of several compounds and therefore the most effective compounds still need to be discovered.

## Data Availability

The datasets used or analyzed during the current study are available from the corresponding author on reasonable request.
